# Syncope in haemodynamically stable and unstable patients with acute pulmonary embolism – Results of the German nationwide inpatient sample

**DOI:** 10.1038/s41598-018-33858-1

**Published:** 2018-10-25

**Authors:** Karsten Keller, Lukas Hobohm, Thomas Münzel, Mir Abolfazl Ostad, Christine Espinola-Klein

**Affiliations:** 1 Center for Thrombosis and Hemostasis (CTH), University Medical Center, Johannes Gutenberg University Mainz, Mainz, Germany; 2 Center for Cardiology – Cardiology I, University Medical Center, Johannes Gutenberg University Mainz, Mainz, Germany; 3 German Center for Cardiovascular Research (DZHK), partner site Rhine Main, Mainz, Germany

## Abstract

Syncope in pulmonary embolism (PE) could be the first sign of haemodynamic compromise. We aimed to investigate pathomechanisms of syncope and its impact on mortality. For this study, patients (aged ≥ 18years) were selected by screening the German nationwide inpatient sample for PE and stratified included patients by syncope (2011–2014). We analysed predictors of syncope in haemodynamically stable PE. Impact of syncope on in-hospital mortality in haemodynamically stable and unstable PE and benefit of systemic thrombolysis in haemodynamically stable PE with syncope (PE + Syncope) were analyzed. The German nationwide inpatient sample comprised 293,640 (84.9%) haemodynamically stable and 52,249 (15.1%) unstable PE patients; among them 2.3% had syncope. Right ventricular dysfunction (RVD) was a key predictor for syncope. In-hospital mortality-rate was lower in haemodynamically stable (6.4% vs. 7.6%, P < 0.001) and unstable PE + Syncope than in PE−Syncope (48.4% vs. 55.5%, P < 0.001) with reduced risk for in-hospital death in stable (OR 0.68 (95%CI 0.61–0.75), P < 0.001) and unstable (OR 0.69 (95% CI 0.62–0.78), P < 0.001) inpatients independent of age and sex. Haemodynamically stable PE + Syncope patients were more often treated with systemic thrombolysis (3.1% vs. 2.1%, P < 0.001). Systemic thrombolysis was associated with reduced in-hospital mortality in haemodynamically stable PE + Syncope (1.9% vs. 6.6%, P = 0.004) independently of age, RVD and tachycardia (OR 0.30 (95%CI 0.11–0.82), P = 0.019). In conclusion, in-hospital mortality was 6.4% in haemodynamically stable PE + Syncope. Haemodynamically stable PE + Syncope patients were more often treated with systemic thrombolysis and showed a trend to improved survial.

## Introduction

Syncope is defined as a transient loss of consciousness due to transient global cerebral hypoperfusion^1,2^. It is characterized by rapid onset, short duration, followed by spontaneous complete recovery^1,2^ and has to be differentiated from transient loss of consciousness caused by other disorders for example epileptic seizures^1,2^. Often, syncope occurs without warning and the exact duration of consciousness could be rarely estimated^1,2^.

According to current classification, syncope can be neurally caused (i.e., vasovagal, situational, or carotid-sinus-syndrome), mediated by orthostatic hypotension (i.e., drug-induced, due to primary or secondary autonomic failure or due to volume depletion), or could have a cardiovascular origin (i.e., arrhythmias, structural cardiovascular diseases, or pulmonary embolism [PE])^1–5^.

In general, syncope is a common phenomenon in the general population and a frequent reason for patients’ presentation at the emergency departments of the hospitals^1,6–10^. While most potential causes are of benign origin and often self-limited, some etiologies are related to significant morbidity and mortality^3,4,9^. Overall, the most frequently identified causes of syncope were vasovagal in 21.2%, cardio-vascular in 9.5%, and orthostatic in 9.4%, while for 36.6% the underlying cause remained unknown^11^.

Life-threatening causes for syncope are primarely based on cardio-vascular diseases including PE, but also comprising hemorrhage, and subarachnoidal bleeding^9^. PE in syncope patients was reported in-between 0.8 and 17.3%^3,12–15^. In one large observational study including 1,671,944 patients with syncope in 4 different countries, prevalence of syncope was obeserved between 0.2% and 2.1% of the hospitalized patients^16^.

Although PE is a well recognized cause for syncope, the underlying pathomechanisms for syncope during PE are not completely understood^17,18^. It was hypothesized that syncope in the setting of acute PE is mostly caused by a combination of more than one singular underlying pathomechanism^14,19^. The suggested three main mechanisms are: (i) large pulmonary artery embolus with more than 50% occlusion of the pulmonary vascular tree accompanied by right ventricular dysfunction (RVD) and consecutively impaired left ventricular filling, leading to reduction in cardiac output with arterial hypotension and declined cerebral blood flow resulting in syncope/collapse^19,20^; (ii) PE could be associated with arrhythmias^14,19–23^. Particularly, RVD could increase the risk of arrhythmias in acute PE events with concomitant syncope^14,18–20,23^; (iii) PE with occlusion of pulmonary artery bed could trigger a vasovagal reflex (Bezold-Jarisch reflex) resulting in sudden decline of cardiac output, vasodilatation and neuro-cardiogenic syncope^14,18–20,23–26^. In addition three other pathomechanisms could underlie the syncope event, but presumably these are commonly only cofactors of the main three mentioned mechanisms: (iv) hypoxemia caused by ventilation or perfusion abnormalities may be followed by syncope (presumably most commonly in central PE with large arterial emboli)^20^; (v) orthostatic dysfunction triggered by alterations in the peripheral vascular system^4^; and (vi) comorbidities may trigger syncope.

Altghough systemic thrombolysis is recommended for high-risk PE patients without contraindications, but only for selected haemodynamically stable acute PE patients according to the current ESC 2014 guideline^27^, effect of systemic thrombolysis in PE patients with first signs of haemodynamic compromise such as syncope remains unclear.

Thus, we aimed (1^st^) to investigate the underlying pathomechanisms of syncope in haemodynamically stable PE, (2^nd^) to identify outcome predictors of haemodynamically stable PE patients with syncope (PE + syncope), (3^rd^) to evaluate the benefit of systemic thrombolysis in these haemodynamically stable PE patients with syncope and (4^th^) to investigate the impact of syncope on the in-hospital mortality rate of haemodynamically stable and unstable PE patients.

## Patients and Methods

The German nationwide inpatient statistics (Diagnosis related groups [DRG] statistics) of the years 2011–2014 were used for this analysis. Information includes treatment data from all in-patients processed according to DRG system. In Germany, diagnoses of inpatients are coded according to ICD-10-GM (International Classification of Diseases, 10th Revision with German Modification). DRG-coded diagnoses data of all hospital patients are gathered at the Federal Statistical Office in Germany (Statistisches Bundesamt, DEStatis). Overall, in the years 2011–2014, a total of 74.9 million inpatient files and their diagnoses from German hospitals nationwide were registered. In this time-frame the number of hospitals decreased from 2045 in the year 2011 to 1980 in 2014.

Data of haemodynamically stable and unstable PE patients (ICD code I26) stratified for additionally diagnosed syncope (ICD code R55) (source: RDC of the Federal Statistical Office and the Statistical Offices of the federal states, DRG Statistics 2011–2014, own calculations) were analysed. Haemodynamically stable PE was defined as PE patients without shock (ICD code R57), mechanically ventilation (not including the mechanical ventilation during surgery, OPS codes 8–70 and 8–71), and cardio-pulmonary resuscitation (OPS code 8–77) during the in-hospital stay.

First, we compared (respectively in haemodynamically stable and in unstable PE) PE patients with syncope (PE + Syncope) and PE patients without syncope (PE − Syncope) and anaylsed predictors for syncope in haemodynamically stable PE patients.

Secondly, we compared survivors in PE + Syncope with non-survivors and computed predictors of in-hospital death in the PE + Syncope group of haemodynamically stable PE patients, respectively. In addition, we analysed the impact of systemic thrombolysis on the in-hospital mortality rate of all haemodynamically stable PE patients ≥18 years with syncope excluding those patients, who were treated with surgical embolectomy.

### Definitions

Chronic lung diseases were defined as bronchial asthma, chronic obstructive lung disease, pulmonary arterial hypertension or interstitial lung diseases.

Renal insufficiency included diagnosis of all renal insufficiency stages.

Coagulation abnormalities comprised coagulopathies, hemophilia, purpura and bleeding diathesis consisting disseminated intravascular coagulation.

Thrombophilia was defined as anti-thrombin deficiency, protein C and S deficiency, prothrombin gene mutation, and factor V Leiden mutation, and other types of thrombophilia including anti-phospholipid (anti-cardiolipin) syndrome.

Stroke comprised both stroke entities: ischemic and hemorrhagic stroke.

### Study outcomes

The primary endpoint of this study was all-cause death during in-hospital stay (in-hospital death).

### Ethical aspects and study oversight

Since this nationwide inpatient sample did not involve direct access by the investigators to data on individual patients but only to results provided by the Research Data Center, approval by an ethics committee and informed consent were not required according to German law.

### Statistical analysis

Firstly, we compared the groups PE + Syncope and PE − Syncope in haemodynamically stable and unstable PE patients as well as survivors vs. non-survivors in haemodynamically stable PE + Syncope. Descriptive statistics for relevant baseline comparison of both groups were provided with median and interquartile range (IQR), or absolute numbers and corresponding percentages. Continuous variables were tested using the Wilcoxon-Mann-Whitney test. Categorical variables were compared using Fisher’s exact or Chi square test, as appropriate.

Secondly, we performed univariate and multivariate logistic regression analyses to find predictors for syncope in haemodynamically stable PE patients as well as predictors for case-fatality rate in haemodynamically stable PE + Syncope. Multivariate regression models integrated all factors in one model (age, sex, obesity, surgery, cancer, coronary artery disease (CAD), heart failure (HF), chronic lung diseases, essential arterial hypertension, renal insufficiency, diabetes mellitus, coagulation abnormalities, stroke, atrial fibrillation or flutter (AF), vestibular disorders, carotid sinus syndrome, meningioma, pneumonia, sick-sinus-syndrome, ventricular tachycardia caused by re-entry tachycardia and pacemaker or implantable cardioverter defibrillator malfunction). For the risk stratification markers RVD and tachycardia, multivariate logistic regression models were adjusted for the same parameters (adjustment I: age, sex, obesity, surgery, cancer, CAD, HF, chronic lung diseases, essential arterial hypertension, renal insufficiency, diabetes mellitus, coagulation abnormalities, stroke, AF, vestibular disorders, carotid sinus syndrome, meningioma, pneumonia, sick-sinus-syndrome, ventricular tachycardia caused by re-entry tachycardia and pacemaker and implantable cardioverter defibrillator malfunction).

In addition, we provided univariate and multivariate regression models for influence of syncope on in-hospital death in haemodynamically stable and unstable PE patients as well as systemic thrombolysis on case-fatality rate in the group of haemodynamically stable PE + Syncope. The multivariate models were respectively adjusted for age and gender or adjustment I (please see above). Furthermore, we provided an additional multivariate regression modell for the analysis regarding the impact of systemic thrombolysis on case-fatality rate in the group of haemodynamically stable PE + Syncope adjusted for age, RVD and tachycardia.

Cox regressions and Kaplan Meier curves (with log rank test) were used to compare the survival between PE + Syncope vs. PE − Syncope as well as for PE + Syncope patients with additional diagnosed AF vs. without AF respectively related to the in-hospital stay. The Cox regression analysis were performed unadjusted, adjusted for age and gender as well as for adjustment I (as mentioned above).

The software SPSS® (version 20.0; SPSS Inc., Chicago, Illinois, USA) was used for computerised analysis. P values of <0.05 (two-sided) were considered to be statistically significant.

## Results

In total, 345,889 hospitalized patients aged 18 years or older were diagnosed with PE between 2011 and 2014. Among these, 7,936 (2.3%) had a syncope. The large majority (293,640 (84.9%)) of PE patients was haemodynamically stable, while 52,249 (15.1%) showed a haemodynamical instability according to the aformentioned definition.

### Comparison of PE patients with and without syncope

**Figure 1:**
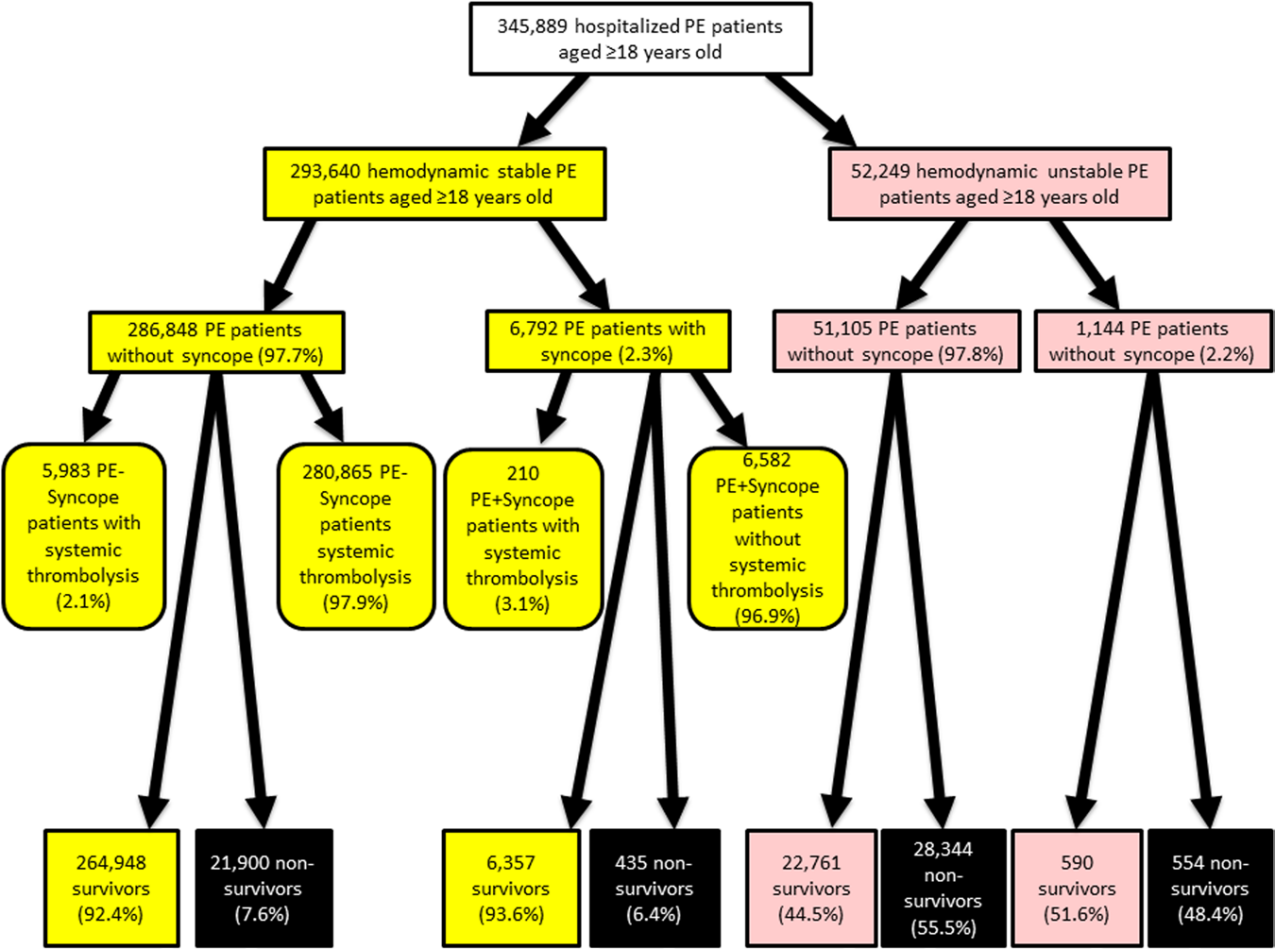
Flow chart of the PE patients with and without syncope.

Focusing on the haemodynamically stable PE patients, 6,792 (2.3%) were additionally diagnosed with syncope, while 286,848 (97.7%) were coded without (see flow chart in Fig. 1). Haemodynamically stable patients + Syncope were in median 4 years older and more often female (57.0%) (Table 1). Suprisingly, PE patients with syncope stayed shorter in-hospital than those without. Typical risk factors for venous thromboembolism (VTE) such as surgery, cancer and thrombophilia were all less frequently observed in PE + Syncope patients compared to PE − Syncope patients. In contrast, cardiovascular diseases and cardiovascular risk factors such as CAD, HF, essential arterial hypertension, diabetes, sick-sinus-syndrome and AF were more prevelant in PE + Syncope group. Renal insufficiency, stroke, deep venous thrombosis (DVT) as well as pneumonia were less often identified in the group PE + Syncope compared to PE − Syncope. The analysed risk stratification markers tachycardia and RVD were more frequently detected in PE + Syncope (Table 1).

**Table 1:** Baseline characteristics, medical history and presentation of the 293,640 haemodynamically stable and 52,249 haemodynamically unstable PE patients aged 18 years and older stratified according presence of syncope.

Parameters	Haemodynamic stable PE patients (n = 293,640; 84.9%)			Haemodynamically unstable PE patients (n = 52,249; 15.1%)		
	PE − Synccope	PE + Syncope	P-value	PE − Synccope	PE + Syncope	P-value
	(n = 286,848; 97.7%)	(n = 6,792; 2.3%)		(n = 51,105; 97.8%)	(n = 1,144; 2.2%)	
Age	72.0 (60.0–80.0)	76.0 (68.0–83.0)	**<0.001**	73.0 (61.0–80.0)	75.0 (67.0–82.0)	**<0.001**
Female gender	154,153 (53.7%)	3,872 (57.0%)	**<0.001**	25,720 (50.3%)	585 (51.1%)	0.591
In-hospital stay (days)	9 (6–14)	7 (10–16)	**<0.001**	12.0 (2.0–26.0)	12.5 (4.0–25.0)	0.182
Obesity	23,314 (8.1%)	601 (8.8%)	**0.032**	5830 (11.4%)	130 (11.4%)	0.963
**VTE risk factors**						
Surgery during in-hospital stay	140,870 (50.9%)	3,148 (46.8%)	**<0.001**	32,285 (63.2%)	682 (59.6%)	**0.014**
Orthopedic surgery during in-hospital stay	10,045 (3.5%)	275 (4.0%)	**0.016**	6,691 (13.1%)	121 (10.6%)	**0.012**
Gastroenterological surgery during in-hospital stay	40,009 (13.9%)	813 (12.0%)	**<0.001**	13,055 (25.5%)	238 (20.8%)	**<0.001**
Cancer	58,618 (20.4%)	858 (12.6%)	**<0.001**	9,436 (18.5%)	175 (15.3%)	**0.006**
**Comorbidities**						
Coronary artery disease	36,280 (12.6%)	1,065 (15.7%)	**<0.001**	9,104 (17.8%)	235 (20.5%)	**0.017**
Heart failure	55,762 (19.4%)	1,574 (23.2%)	**<0.001**	17,814 (34.9%)	446 (39.0%)	**0.004**
Heart failure (NYHA functional class III/IV)	25,992 (9.1%)	644 (9.5%)	0.233	10,142 (19.8%)	224 (19.6%)	0.849
Chronic lung disease	50,878 (17.7%)	1,091 (16.1%)	**<0.001**	11,906 (23.3%)	271 (23.7%)	0.757
Essential arterial hypertension	130,612 (45.5%)	3,417 (50.3%)	**<0.001**	21,118 (41.3%)	536 (46.9%)	**<0.001**
Renal insufficiency	54,267 (18.9%)	1,836 (27.0%)	**<0.001**	18,209 (35.6%)	463 (40.5%)	**0.001**
Diabetes mellitus	51,440 (17.9%)	1,412 (20.8%)	**<0.001**	12,988 (25.4%)	294 (25.7%)	0.827
Coagulation abnormalities	21,711 (7.6%)	481 (7.1%)	0.133	10,749 (21.0%)	227 (19.8%)	0.328
Thrombophilia	5,293 (1.8%)	68 (1.0%)	**<0.001**	557 (1.1%)	18 (1.6%)	0.121
Stroke	5,876 (2.0%)	111 (1.6%)	**0.017**	3,095 (6.1%)	60 (5.2%)	0.255
Atrial fibrillation or flutter	39,427 (13.7%)	1,179 (17.4%)	**<0.001**	12,179 (23.8%)	307 (26.8%)	**0.018**
Vestibular disorder	630 (0.2%)	29 (0.4%)	**<0.001**	55 (0.1%)	4 (0.3%)	**0.040**
Carotid-sinus-syndrome	9 (0.003%)	4 (0.1%)	**<0.001**	6 (0.01%)	0 (0.0%)	**1.000**
Meningioma	535 (0.2%)	18 (0.3%)	0.140	169 (0.3%)	3 (0.3%)	**1.000**
Pneumonia	64,772 (22.6%)	1,091 (16.1%)	**<0.001**	16,187 (31.7%)	323 (28.2%)	**0.014**
Sick-sinus-syndrome	799 (0.3%)	97 (1.4%)	**<0.001**	265 (0.5%)	28 (2.4%)	**<0.001**
Ventricular tachycardia caused by reentry tachycardia	—	—	0.106	—	—	**0.032**
Pacemaker or internal cardiac defibrillator malfunction	108 (0.04)	15 (0.2%)	**<0.001**	56 (0.1%)	4 (0.3%)	**0.042**
Deep venous thrombosis	114,620 (40.0%)	2,388 (35.2%)	**<0.001**	9,029 (17.7%)	217 (19.0%)	0.254
**Risk stratification**						
Tachycardia	3,637 (1.3%)	167 (2.5%)	**<0.001**	2,472 (4.8%)	76 (6.6%)	**0.005**
Right ventricular dysfunction	64,713 (22.6%)	2,021 (29.8%)	**<0.001**	27,224 (53.3%)	660 (57.7%)	**0.003**
**Treatment**						
Systemic thrombolysis	5,983 (2.1%)	210 (3.1%)	**<0.001**	9,050 (17.7%)	215 (18.8%)	0.342
Surgical embolectomy	134 (0.05%)	2 (0.03%)	0.775	319 (0.6%)	7 (0.6%)	1.000
Transfusion of blood constituents	21,975 (7.7%)	542 (8.0%)	0.329	17,922 (35.1%)	351 (30.7%)	**0.002**
**Outcome**						
All cause in-hospital death	21,900 (7.6%)	435 (6.4%)	**<0.001**	28,344 (55.5%)	554 (48.4%)	**<0.001**

In case of blank fields, the absolute numbers are very small and were not provided by the Federal Statistical Office of Germany due to secrecy reasons, nevertheless the statistical testing could be performed.

Abbreviations: PE indicates for pulmonary embolism; VTE, venous thromboembolism; NYHA, New York Heart Association.

Indipendent predictors for syncope in haemodynamically stable PE patients were age, obesity, renal insufficiency, heart-rhythm-disturbances (sick-sinus-syndrome, tachycardia and pacemaker or implantable cardioverter defibrillator malfunction), RVD and last but not least carotid-sinus-syndrome (Table 2).

**Table 2:** Predictors of syncope in patients with pulmonary embolism aged 18 years and older (uni-variate and multi-variate logistic regression models).

Parameters	PE patients aged 18 years and older (n = 293,640; 6,792 patients with syncope [2.3%])			
	Univariate regression model		Multi-variate regression model	
	OR (95% CI)	P-value	OR (95% CI)	P-value
Age	1.024 (1.022–1.026)	**<0.001**	1.020 (1.018–1.022)	**<0.001**
Female gender	1.142 (1.088–1.199)	**<0.001**	0.973 (0.925–1.022)	0.282
Obesity	1.097 (1.008–1.194)	**0.032**	1.138 (1.043–1.241)	**0.004**
**VTE risk factors**				
Surgery during in-hospital stay	0.911 (0.868–0.956)	**<0.001**	0.958 (0.912–1.006)	0.088
Cancer	0.563 (0.524–0.605)	**<0.001**	0.587 (0.545–0.632)	**<0.001**
**Comorbidities**				
Coronary artery disease	1.284 (1.202–1.372)	**<0.001**	1.036 (0.967–1.110)	0.318
Heart failure	1.250 (1.181–1.324)	**<0.001**	0.987 (0.927–1.050)	0.671
Chronic lung disease	0.888 (0.831–0.948)	**<0.001**	0.775 (0.725–0.828)	**<0.001**
Essential arterial hypertension	1.211 (1.154–1.271)	**<0.001**	1.021 (0.972–1.073)	0.407
Renal insufficiency	1.588 (1.504–1.676)	**<0.001**	1.294 (1.220–1.372)	**<0.001**
Diabetes mellitus	1.201 (1.132–1.275)	**<0.001**	1.025 (0.954–1.090)	0.433
Coagulation abnormalities	0.931 (0.847–1.022)	0.134	1.017 (0.925–1.118)	0.726
Stroke	0.794 (0.657–0.960)	**0.017**	0.716 (0.592–0.866)	**0.001**
Atrial fibrillation or flutter	1.318 (1.237–1.405)	**<0.001**	1.025 (0.958–1.097)	0.474
Vestibular disorder	1.948 (1.342–2.829)	**<0.001**	1.641 (1.128–2.389)	**0.010**
Carotid-sinus-syndrome	18.781 ((5.782–61.000)	**<0.001**	13.062 (3.816–44.707)	**<0.001**
Meningioma	1.422 (0.889–2.276)	0.142	1.398 (0.872–2.240)	0.164
Pneumonia	0.656 (0.615–0.700)	**<0.001**	0.661 (0.619–0.706)	**<0.001**
Sick-sinus-syndrome	5.187 (4.196–6.413)	**<0.001**	3.869 (3.110–4.812)	**<0.001**
Ventricular tachycardia caused by reentry tachycardia	3.840 (0.903–16.334)	0.069	4.037 (0.939–17.358)	0.061
Pacemaker or internal cardiac defibrillator malfunction	5.876 (3.422–10.090)	**<0.001**	3.425 (1.953–6.008)	**<0.001**
**Risk stratification parameters**				
Tachycardia	1.963 (1.678–2.297)	**<0.001**	1.870 (1.594–2.194)	**<0.001**
Right ventricular dysfunction	1.454 (1.379–1.533)	**<0.001**	1.347 (1.276–1.421)	**<0.001**

Abbreviations: PE indicates for pulmonary embolism; VTE, venous thromboembolism.

**Figure 2:**
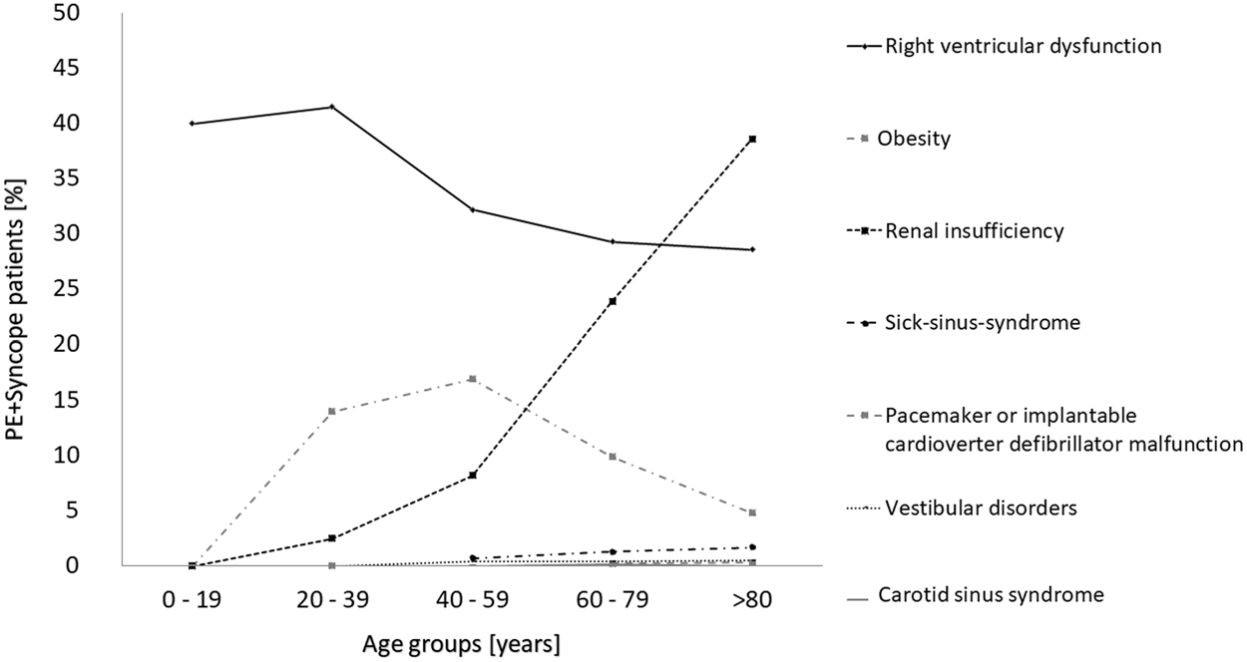
Frequency of factors associated with syncope in PE patients stratified for age-groups.

Remarkably, when focusing on the independent predictors for syncope mentioned above and their prevalence in the PE + syncope patients, RVD and carotid-sinus-syndrome decreased in their frequency in PE + Syncope group with growing age, whereas renal insufficiency and sick-sinus-syndrome increased in their prevalence with older age (Fig. 2). While RVD and renal insufficiency might be key factors in syncope development accompanied by high frequency, the other comorbidities such as heart-rhythm-disturbances (sick-sinus-syndrome, tachycardia and pacemaker or implantable cardioverter defibrillator malfunction), vestibular disorders and carotid-sinus-syndrome are less common in these patients. Especially, RVD with consistently a frequency of >25% in all age-groups is of outstanding importance regarding the development of syncope and renal insufficiency might have its consideration especially in older age as an contributing factor.

In haemodynamically unstable PE patients, syncope was diagnosed in 1,144 (2.2%) patients aged 18 years or older (see flow chart in Fig. 1). Patients with syncope were in median 2 years older. Cardiovascular diseases such as CAD, HF and AF were, consistently to the aforementioned haemodynamically stable PE patients, more common in PE + Syncope than in PE − Syncope. In addition, RVD, tachycardia as well as sick sinus syndrome were more often found in PE + Syncope (Table 1).

### In-hospital mortality

The in-hospital mortality rate was distinctly higher in haemodynamically unstable PE patients in comparison to haemodynamically stable patients (55.3% vs. 7.6%).

Unexpectedly, the mortality rate was lower in PE + Syncope compared to PE − Syncope (haemodynamically stable PE: 435 [6.4%] vs. 21,900 [7.6%], P < 0.001; haemodynamically unstable PE: 554 [48.4%] vs. 28,344 [55.5%], P < 0.001) (Table 1). In the crude logistic regression model, syncope was associated with a lower risk for in-hospital death in haemodynamically stable (OR 0.83 (0.75–0.91), P < 0.001) as well as for unstable patients (OR 0.75 (95%CI 0.67–0.85), P < 0.001). These results remained stable after adjustment for age and sex (haemodynamically stable patients: OR 0.68 (0.61–0.75), P < 0.001; haemodynamically unstable patients: OR 0.69 (95%CI 0.62–0.78), P < 0.001) as well as after adjustment for age, sex and comorbidities (haemodynamically stable patients: OR 0.73 (0.66–0.80), P < 0.001; haemodynamically unstable patients: OR 0.65 (95%CI 0.57–0.73), P < 0.001).

**Figure 3:**
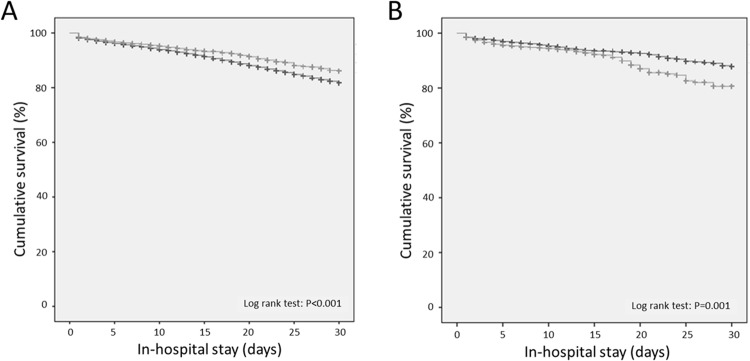
(**A**) Cumulative survival in PE patients with (light gray line) and without (dark gray line) syncope related to the in-hospital stay (the observation period for the Kaplan-Meier plot was limited to the first 30 days). (**B**) Cumulative survival in PE patients with syncope with additional AF (light gray line) and without additional AF (dark gray line) related to the in-hospital stay (the observation period for the Kaplan-Meier plot was limited to the first 30 days).

Also the Cox regression model for cumulative survival related to the in-hospital stay adjusted for age and gender highlighted a survival benefit for haemodynamically stable PE + Syncope patients (HR 0.72 (0.68–0.77), P < 0.001) illustrated in the Kaplan Meier curves (Fig. 3A). The result remained stable after adjustment for age, gender and all comorbidities (HR 0.69 (0.63–0.76), P < 0.001).

When comparing haemodynamically stable PE + Syncope non-survivors vs. survivors, non-survivors were in median 7 years older, leaner and had more often comorbidities such as cancer, HF, AF, renal insufficiency, coagulation abnormalities, as well as pneumonia. Among the investigated risk stratification markers, only RVD was more frequently detected in the non-survivors (Table 3).

**Table 3:** Baseline characteristics, medical history and presentation of the 6,792 haemodynamically stable PE patients aged 18 years and older with presence of syncope stratified according in-hospital death.

Parameters	PE + Syncope survivors (n = 6,357; 93.6%)	PE + Syncope non-survivors (n = 435; 6.4%)	P-value
Age	76.0 (67.0–83.0)	83.0 (75.0–87.0)	**<0.001**
Female gender	3,621 (57.0%)	251 (57.7%)	0.766
In-hospital stay (days)	10 (7–16)	5 (2–13)	**<0.001**
Obesity	578 (9.1%)	23 (5.3%)	**0.007**
**VTE risk factors**			
Surgery during in-hospital stay	3,021 (47.5%)	157 (36.1%)	**<0.001**
Orthopedic surgery during in-hospital stay	252 (4.0%)	23 (5.3%)	0.112
Gastroenterological surgery during in-hospital stay	770 (12.1%)	43 (9.9%)	0.166
Cancer	738 (11.6%)	120 (27.6%)	**<0.001**
**Comorbidities**			
Coronary artery disease	981 (15.4%)	84 (19.3%)	**0.031**
Heart failure	1,416 (22.3%)	158 (36.3%)	**<0.001**
Heart failure (NYHA functional class III/IV)	552 (8.7%)	92 (21.1%)	**<0.001**
Chronic lung disease	1,012 (15.9%)	79 (18.2%)	0.218
Essential arterial hypertension	3,221 (50.7%)	196 (45.1%)	**0.024**
Renal insufficiency	1,661 (26.1%)	175 (40.2%)	**<0.001**
Diabetes mellitus	1,304 (20.5%)	108 (24.8%)	**0.032**
Coagulation abnormalities	435 (6.8%)	46 (10.6%)	**0.003**
Stroke	100 (1.6%)	11 (2.5%)	0.166
Atrial fibrillation or flutter	1,066 (16.8%)	113 (26.0%)	**<0.001**
Vestibular disorder	29 (0.5%)	0 (0.0%)	0.257
Carotid-sinus-syndrome	4 (0.1%)	0 (0.0%)	1.000
Meningioma	—	—	1.000
Pneumonia	1,000 (15.7%)	91 (20.9%)	**0.004**
Sick-sinus-syndrome	93 (1.5%)	4 (0.9%)	0.528
Ventricular tachycardia caused by reentry tachycardia	—	—	0.124
Pacemaker oder internal cardiac defibrillator malfunction	15 (0.2)	0 (0.0%)	0.619
Deep venous thrombosis	2,321 (36.5%)	67 (15.4%)	**<0.001**
**Risk stratification**			
Tachycardia	156 (2.5%)	11 (2.5%)	0.873
Right ventricular dysfunction	1,826 (28.7%)	195 (44.8%)	**<0.001**
**Treatment**			
Systemic thrombolysis	206 (3.2%)	4 (0.9%)	**0.004**
Surgical embolectomy	2 (0.03%)	0 (0.0%)	1.000
Transfusion of blood constituents	481 (7.6%)	61 (14.0%)	**<0.001**

In case of blank fields, the absolute numbers are very small and were not provided by the Federal Statistical Office of Germany due to secrecy reasons, nevertheless the statistical testing could be performed.

Abbreviations: PE indicates for pulmonary embolism; VTE, venous thromboembolism; NYHA, New York Heart Association.

Independent predictors of in-hospital death in haemodynamically stable PE patients with syncope were age, cancer, HF, renal insufficiency, coagulation abnormalities, AF, pneumonia and RVD, whereas essential arterial hypertension and surgery during in-hospital stay were accompanied by lower mortality (Table 4).

**Table 4:** Predictors of all-cause in-hospital death in haemodynamically stable PE patients with syncope aged 18 years and older (uni-variate and multi-variate logistic regression model).

Parameters	PE + Syncope aged 18 years and older (n = 6,792 patients with syncope; 435 patients [6.4%] died in-hospital)			
	Univariate regression model		Multi-variate regression model	
	OR (95% CI)	P-value	OR (95% CI)	P-value
Age	1.058 (1.047–1.069)	**<0.001**	1.057 (1.045–1.070)	**<0.001**
Female gender	1.030 (0.846–1.254)	0.766	1.004 (0.812–1.240)	0.972
Obesity	0.558 (0.364–0.857)	**0.008**	0.754 (0.482–1.179)	0.216
**VTE risk factors**				
Surgery during in-hospital stay	0.624 (0.510–0.763)	**<0.001**	0.526 (0.424–0.654)	**<0.001**
Cancer	2.901 (2.319–3.628)	**<0.001**	4.218 (3.300–5.393)	**<0.001**
**Comorbidities**				
Coronary artery disease	1.311 (1.024–1.680)	**0.032**	1.163 (0.893–1.513)	0.262
Heart failure	1.990 (1.623–2.441)	**<0.001**	1.511 (1.211–1.887)	**<0.001**
Chronic lung disease	1.172 (0.910–1.509)	0.218	0.986 (0.755–1.287)	0.915
Essential arterial hypertension	0.798 (0.657–0.971)	**0.024**	0.689 (0.562–0.846)	**<0.001**
Renal insufficiency	1.903 (1.559–2.323)	**<0.001**	1.271 (1.021–1.581)	**0.032**
Diabetes mellitus	1.280 (1.021–1.604)	**0.032**	1.152 (0.907–1.462)	0.247
Coagulation abnormalities	1.610 (1.168–2.219)	**0.004**	1.664 (1.183–2.341)	**0.003**
Stroke	1.623 (0.864–3.049)	0.132	1.743 (0.896–3.389)	0.102
Atrial fibrillation or flutter	1.742 (1.392–2.180)	**<0.001**	1.322 (1.041–1.678)	**0.022**
Vestibular disorder	Not calculable		Not calculable	
Carotid-sinus-syndrome	Not calculable		Not calculable	
Meningioma	0.859 (0.114–6.472)	0.883	0.836 (0.103–6.781)	0.867
Pneumonia	1.417 (1.114–1.803)	**0.005**	1.341 (1.041–1.728)	**0.023**
Sick-sinus-syndrome	0.625 (0.229–1.709)	0.360	0.592 (0.211–1.657)	0.318
Ventricular tachycardia caused by reentry tachycardia	14.645 (0.914–234.539)	0.058	8.271 (0.451–151.552)	0.154
Pacemaker or internal cardiac defibrillator malfunction	Not calculable		Not calculable	
**Risk stratification parameters**				
Tachycardia	1.031 (0.555–1.916)	0.922	0.886 (0.452–1.736)	0.724
Right ventricular dysfunction	2.016 (1.656–2.454)	**<0.001**	2.136 (1.733–2.633)	**<0.001**

Abbreviations: PE indicates for pulmonary embolism; VTE, venous thromboembolism.

Notably, the Cox regression model for cumulative survival (related to the in-hospital course) revealed, that the presence of AF in haemodynamically stable PE patients with syncope increased the risk for in-hospital death when adjusted for age and gender (HR 1.11 (1.09–1.14), P < 0.001), also confimed in the Kaplan Meier curves (Fig. 3B) and the corresponding log-rank test; but the result remained not significant associated after adjustment for age, gender and all comorbidities in the same Cox regression model (HR 1.20 (0.96–1.50), P = 0.107).

### Systemic thrombolysis in adult haemodynamically stable PE patients with syncope

Interestingly, the PE + Syncope group was more often treated with systemic thrombolysis and had a lower case-fatality rate (Table 1). Consequently, also haemodynamically stable PE + Syncope survivors were more often treated with systemic thrombolysis, while non-survivors needed more often transfusion of blood constituents (Table 3).

In total 3.1% of the haemodynamically stable PE patients (210/6,792) with syncope were treated with systemic thrombolytic therapy. Mortality rate was significantly lower in PE + Syncope patients treated with systemic thrombolysis in comparison to those without (4/210 [1.9%] vs. 431/6582 [6.6%], P = 0.004). Remarkably, PE + Syncope patients, who were treated with systemic thrombolysis showed a lower risk for in-hospital death in the univariate logistic regression model (OR 0.28 (0.10–0.75), P = 0.011). After adjustment for age, RVD and tachycardia this association remained still significant (OR 0.30 (0.11–0.82), P = 0.019). In the multivariate logistic regression model adjusted for age and sex (OR 0.43 (0.16–1.16), P = 0.094) as well as after adjustment for age, sex and comorbidities (OR 0.42 (0.15–1.17), P = 0.096) the association demonstrated a trend towards lower risk for in-hospital death in PE + Syncope patients by the administration of systemic thrombolysis with a borderline significance level, respectively.

As expected, haemodynamically stable PE + Syncope patients, who were treated with systemic thrombolysis, had a higher rate of intracerebral bleeding (3 [1.4%] vs. 19 [0.3%], P = 0.029), but not of subarachnoid bleeding (P = 0.118) and gastro-intestinal bleeding (P = 1.000).

## Discussion

In the large cohort of the German nationwide inpatient sample, we aimed to investigate the pathomechanisms of syncope in haemodynamically stable PE patients, to identify outcome predictors of haemodynamically stable PE patients (aged ≥ 18 years) with syncope and to evaluate the benefit of systemic thrombolysis in these patients.

The key findings of our analysis could be summarized as follows: 1^st^) Factors independently associated with syncope development in haemodynamically stable PE patients were RVD, carotid-sinus-syndrome, heart-rhythm-disturbances, obesity and renal impairment. 2^nd^) Syncope in haemodynamically stable and unstable PE patients was accompanied by better survival. 3^rd^) Independent predictors of in-hospital death in haemodynamically stable PE patients with syncope group were age, cancer, HF, renal insufficiency, coagulation abnormalities, AF, pneumonia and RVD. 4^th^) Haemodynamically stable PE patients with syncope were more often treated with systemic thrombolysis accompanied by a trend to improved survival.

The symptom syncope is a common phenomenon in the general population^1,6^. Up to 1/3 of the general population develop at least one syncope during life-time^6–8^ and between 1% and 3% of the referrals in the emergency departments of the hospitals are for syncopes^1,8–10,28^. Although syncope in general is mostly accompanied by a low mortality, contrastly, syncope in PE patients is attended by high mortality^9,11^.

In total, 2.3% of the PE patients of the German nationwide inpatients sample had a coded syncope, which is within the reported range of other studies (0.8 and 8.1%^12–15,28^). As expected and in accordance with literature^4,14,18–20,23^, the results of our study highlighted that cardiac adaptations due to PE such as RVD were associated with development of syncope. RVD had a high prevalence in all age-subgroups from the young to the old. PE with larger occlusions in the pulmonary artery tree resulting in RVD^14,18–20,23^ as well as hypoxia^20^ and might also be accompanied by arrhythmias^14,19–23^. RVD and arrhythmias can cause syncope due to a reduction in cardiac output with arterial hypotension and declined cerebral blood flow^19,20^; another underlying mechanism might be the vasovagal Bezold-Jarisch reflex triggered by occlusion of pulmonary artery bed followed by sudden decline of cardiac output, vasodilatation and neuro-cardiogenic syncope^14,18–20,23–26^. In addition, hypoxemia could lead to cerebral undersupply with oxygen. Moreover, comorbidities might promote syncope such as renal insufficiency, maybe favoured by hypotension and cardio-renal adaptations.

Beside these factors, which seem to cause a syncope based on haemodynamic compromise, reduced cardiac output or hypoxemia driven directly by the PE event, we identified a second patient group of haemodynomically stable PE patients with syncope suffering of typical comorbidities, in whom the comorbidities might be responsible for syncope development and therefore syncope may underlie another pathomechanism in this patient group. In these haemodynamically stable patients, we observed comorbidities such as vestibular disorders and carotid-sinus syndrome, which are typical benign reasons for occurrence of syncope also in individuals without PE^1,4,29^. Although the syncope occurred in temporal context with the PE event, the underlying pathomechanism for development of the syncope might be in the majority of these patients (of this second group) different and not the haemodynamic compromise and/or the hypoxemia^4^. This hypothesis is supported by differences regarding outcome: while the syncope events, which are directly based on haemodynamic compromise and hypoxemia (due to the PE) are affected by poorer prognosis in haemodynamically stable PE patients, PE patients with the comorbidities of vestibular disorders and carotid-sinus revealed a better survival indicating for heterogenous pathomechanisms of syncope also in PE patients. These findings might justify also in PE patients a search for specific reasons of syncope beside the diagnosis of PE and identification of RVD, especially in PE patients with low thrombus burden (segmental or subsegmental PE) and without identified RVD or myocardial injury^4,29^.

Although the diagnostic value of transthoracic echocardiography in diagnostic approach of syncope was considered in general to be low^30^, our study results emphasize the importance of transthoracic echocardiography in patients with syncope as well as PE patients. Presence of RVD in syncope patients should encourage to induce further examinations to detect a presumable PE, and RVD in PE patients, in whom RVD is an independent predictor of in-hospital death (confirmed in our study), the detection of RVD should guide the physicians to monitor the patients more intensively and in cases of PE patients with syncope, to consider thrombolytic therapy according to the recommendations of the current ESC guidelines^27,31^.

Interestingly, overall, syncope was in haemodynamically stable and unstable PE patients in our study accompanied by better survival. This result seems, on the first look, to be in contrast with the literature, in which syncope was described as haemodynamic decompensation with increased mortality^27,31,32^; but most of these studies included haemodynamically stable as well as unstable PE patients and did not distinguish between both patient subgroups. Similarly to our study results, some studies reported a non-inferior outcome of PE patients with syncope compared to those without^32,33^. Lee *et al*.^32^ reported that syncope did not influence the short-term prognosis of patients with PE^32^ and Roncon *et al*.^33^ described that a difference in survival was only identified in haemodynamically unstable PE patients, but not for haemodynamically stable patients^33^. There is strong evidence that syncope lead to better monitoring and earlier treatment in patients with syncope (as a sign of impending shock). More intensive monitoring and more aggressive, early administered, treatments might lead to better survival in PE patients with syncope in our study. Nevertheless, particularly, the better survival of haemodynamically unstable PE patients with syncope in our study might surprise, but it has to be considered that syncope as a symptom of impending shock or in cases, in which syncope temporally precede cardio-pulmonary resuscitation, might not be additively coded and might therefore be under-coded in the nationwide-inpatient sample with a distortion and bias regarding better survival of haemodynamically unstable PE patients with syncope. Therefore, these findings have to be interpreted with caution.

Although the reasons of the better survival in PE patients, who did not develop heamodynamically instability, could not fully elucidated, it has to be assumed that in haemodynamically stable PE patients with syncope systemic thrombolytic treatment might be considered earlier and more commonly. This may be one further explanation of our study results regarding a better survival in haemodynamic stable PE patients with syncope in comparison to those patients without syncope.

Although systemic thrombolysis is not the treatment of choice for most cases of haemodynamically stable acute PE patients according to the ESC 2014 guideline^27^, in patients with haemodynamic compromise, including syncope, thrombolytic therapy could and should be considered as treatment strategy^27^. In accordance with these recommendations^27^, haemodynamically stable PE patients with syncope in Germany were more often treated with systemic thrombolysis in comparison to haemodynamically stable PE patients without. Remarkably, PE patients with syncope, who were treated with systemic thrombolysis showed a survival benefit with mortality reduction (4/210 [1.9%] vs. 431/6582 [6.6%], P = 0.004). The lower in-hospital mortality risk was confirmed in the univariate regression analysis and was also shown independently of age, RVD and tachycardia. In the multivariate regression analysis adjusted for sex and age as well as adjusted for age, sex and additional adjustment for comorbidities this association demonstrated still a trend towards significance. Our results are partly in acocordance with the results of Stein *et al*.^34^, who reported that thrombolytic therapy in unstable PE patients saves lives, but is underused^34^. It is well established that thrombolytic treatment in acute PE restores pulmonary perfusion more rapidly than anticoagulation alone and is connected with a prompt reduction in pulmonary artery pressure and resistance, resulting in a concomitant improvement of right ventricular function^27^. Our results support the beneficial effect of systemic thrombolysis in haemodynamic compromised patients without shock or cardio-pulmonary resusciatation and may indicate for an early treatment with systemic thrombolysis in the beginning of haemodynamic compromise not waiting for complete formation of shock (signs) in selected PE patients. However, it should not be forgotten, that a thrombolytic treatment carries a relevant risk of major bleeding, including intracranial haemorrhage^27,31^. While in our evaluation the rate of intracerebral bleeding events were only slightly elevated in the PE + Syncope patients treated with systemic thrombolysis in comparison to those without, subarachnoid and gastro-inetestinal beleeding were not significantly elevated.

## Limitations

There are some limitations regarding our study that require consideration. Firstly, the study results are based on ICD and OPS discharge codes of hospitalized patients, which might lead to incomplete data due to under-reporting/under-coding. Secondly, the exact time point of haemodynamic instability (i.e. whether it was present on admission or become apparent as a complication during the in-hospital stay) could not be identified. Thirdly, important clinical data such as information about computed tomography with degree of the severity of the PE, cardiac troponin plasma concentrations, echocardiographic results about singular parameters of RVD or concomitant medications were not available. Therefore, we focused on the hard endpoint of in-hospital death.

## Conclusions

We found a mortality of 6.4% in haemodynamicall stable PE patients with syncope. RVD and heart-rhythm-disturbances seem to play an important role in syncope development. In fact, RVD, cancer as well as AF increased the case-fatality rate of PE + Syncope patients. Haemodynamically stable PE + Syncope patients were more often treated with systemic thrombolysis and administration of systemic thrombolysis in PE + Syncope patients was accompanied by a trend to an improved survial.
